# Implications of Changes to the Federal Funding Landscape for Geriatrics Training Programs

**DOI:** 10.1093/geroni/igaf122.4140

**Published:** 2025-12-31

**Authors:** Apoorva Rangan

**Affiliations:** Stanford University, Los Altos Hills, California, United States

## Abstract

This poster session will describe the history of federal Geriatrics workforce development programs and the implications of proposed cuts to the Geriatrics Workforce Enhancement Programs (GWEPs) and Geriatrics Academic Career Awards (GACAs) on the nation’s capacity to train clinicians in age-friendly care. The 2008 Institute of Medicine report Retooling for an Aging America identified clinical complexity, ageist stereotypes, lack of mentors, and financial disincentives as barriers to geriatrics workforce growth¹. In response, GWEPs (launched in 2015) and GACAs (re-established in 2019 after elimination in 2015) were established to embed interprofessional geriatrics curricula, support early-career educators and researchers, and fortify training sites in underserved settings.² These programs received top-line funding of $42 million in fiscal year 2024, faced severe cuts with top-line funding of $28 million in fiscal year 2025, and have uncertain funding this year.3, 4 Drawing on recent National Center for Health Workforce Analysis workforce projections, American Board of Medical Specialties Board Certification Report, American Geriatrics Society policy materials, and HHS’ Tracking Accountability in Government Grants System (TAGGS), this session will explore how elimination of these federal investments may: 1) interrupt crucial interprofessional training partnerships; 2) erode a critical pipeline of clinician–educators by withdrawing protected time and mentorship; and 3) exacerbate health disparities by constricting age-friendly care capacity for low-income and rural older people. Finally, this session will engage participants in policy recommendations to preserve and expand these programs’ funding, ensuring a sustainable geriatrics workforce equipped to meet the complex needs of all of us as we age.

